# Oncogenic Dysregulation of Circulating Noncoding RNAs: Novel Challenges and Opportunities in Sarcoma Diagnosis and Treatment

**DOI:** 10.3390/cancers14194677

**Published:** 2022-09-26

**Authors:** Lidia Chellini, Ramona Palombo, Veronica Riccioni, Maria Paola Paronetto

**Affiliations:** 1 Laboratory of Molecular and Cellular Neurobiology, IRCCS Fondazione Santa Lucia, 00143 Rome, Italy; 2 Department of Movement, Human and Health Sciences, Università degli Studi di Roma “Foro Italico”, Piazza Lauro de Bosis, 15, 00135 Rome, Italy

**Keywords:** noncoding RNAs, sarcoma, circulating RNAs

## Abstract

**Simple Summary:**

Body fluids contain different classes of RNA molecules such as protein-coding messenger RNAs (mRNA) and noncoding RNAs, including microRNAs (miRNAs), long noncoding RNAs (lncRNAs) and circular RNAs (circRNAs). These circulating RNAs can travel naked or packed into extracellular vesicles and display valuable potential as non-invasive biomarkers of sarcoma malignancy. In this review, we summarize current knowledge on the possible functions of these circulating RNAs and discuss their possible exploitation as novel markers to improve sarcoma diagnosis and prognosis. Despite the recent advance in technological tools have improved protocols for the extraction and detection of circulating RNA, many aspects related to the biology of these molecules remain to be elucidated. In particular, the lack of standardization in the assessment of these markers makes difficult their adoption into clinical practice.

**Abstract:**

Sarcomas comprise a heterogeneous group of rare mesenchymal malignancies. Sarcomas can be grouped into two categories characterized by different prognosis and treatment approaches: soft tissue sarcoma and primary bone sarcoma. In the last years, research on novel diagnostic, prognostic or predictive biomarkers in sarcoma management has been focused on circulating tumor-derived molecules as valuable tools. Liquid biopsies that measure various tumor components, including circulating cell-free DNA and RNA, circulating tumor cells, tumor extracellular vesicles and exosomes, are gaining attention as methods for molecular screening and early diagnosis. Compared with traditional tissue biopsies, liquid biopsies are minimally invasive and blood samples can be collected serially over time to monitor cancer progression. This review will focus on circulating noncoding RNA molecules from liquid biopsies that are dysregulated in sarcoma malignancies and discuss advantages and current limitations of their employment as biomarkers in the management of sarcomas. It will also explore their utility in the evaluation of the clinical response to treatments and of disease relapse. Moreover, it will explore state-of-the-art techniques that allow for the early detection of these circulating biomarkers. Despite the huge potential, current reports highlight poor sensitivity, specificity, and survival benefit of these methods, that are therefore still insufficient for routine screening purposes.

## 1. Introduction

Sarcomas are a large heterogenous group of malignancies which develop from cells of mesenchymal origin. They are classified into two main groups, depending on the tissue where arising [1]. Bone sarcomas originate from bone and mainly include osteosarcoma (OS), Ewing sarcoma (ES) and chondrosarcoma. Soft tissue sarcomas consist of a wide range of histological subtypes, which develop from connective tissue such as fat, muscle, tendons and blood vessels [1]. They include leiomyosarcoma (LMS), liposarcoma (LS), rhabdomyosarcoma (RMS), synovial sarcoma (SS), malignant peripheral nerve sheath tumor (MPNST), and undifferentiated pleomorphic sarcoma (UPS) [1,2]. To date, surgical resection, radiotherapy and chemotherapy are the common treatments for sarcomas [2]. In recent years, multiagent chemotherapy strategies such as the combination of doxorubicin and cisplatin have significantly improved the prognosis of patients with certain sarcoma type. However, in a large part of patients these treatments failed, leading to tumor recurrence, development of metastasis and acquisition of drug resistance [2,3,4]. Therefore, a better understanding of the complex mechanisms of sarcoma pathogenesis is crucial for identifying effectors involved in chemotherapy resistance and metastasis development, defining novel prognostic biomarkers able to predict progression of disease and improving prognosis.

In the last few decades, a significant progress has been made in knowledge and development of liquid-based biopsy techniques [5]. Liquid biopsy is a very simple and minimal invasive procedure, without complication risks, thus representing a valuable alternative to surgical biopsies. The main source of information obtained by liquid biopsy originates from tumor cells, cell-free nucleic acids, exosomes, and metabolites, released and circulating in body fluids such as plasma, serum, saliva, cerebral spinal and urine. Liquid biopsies provide a specific representation of tumor, allowing to monitor tumor dynamics, to characterize primary and recurrent tumors, to predict treatment response, picturing cancer relapse and metastasis [5]. Among cell-free nucleic acids, great attention was focused on circulating tumor DNA (ctDNA), which consists of small pieces of DNA, usually less than 200 nucleotides [6], present in the body fluids and reflecting the entire tumor genome [6]. ctDNA detection was used to study different clinical parameters, such as tumor volume and stage, as well as to monitoring minimal residual disease, response, resistance to therapies and tumor evolution [7]. Although easily detected in aggressive tumors, ctDNAs are present at low levels in sarcoma, making difficult their detection at times of lower disease burden [8]. Moreover, most ctDNA assays are designed to detect recurrent mutations, while pediatric sarcomas show a small number of recurrent mutations and are instead characterized by translocations and copy-number changes. To date, only a small number of studies managed to dissect different subtypes of sarcomas by analyzing ctDNA [9,10]. In contrast, circulating noncoding RNAs are recently emerging as key mediators in the regulation of gene expression profile of tumor cells. This function is reflected also in the body fluids, where they show different expression patterns in cancer patients and healthy subjects, thus representing ideal candidates in blood-based diagnostics [11]. This characteristic provides an advantage over ctDNA allowing to discriminate different noncoding RNA expression profiles, associated with tumor stages or responses to therapies, due to transcriptional changes, not detectable through ctDNA analysis. At present, both ctDNA and circulating noncoding RNAs show several advantages making them promising tools in sarcoma diagnosis and prognosis as biomarkers, accompanied by as many limits to their clinical application. Therefore, the analysis of both types of circulating nucleic acids could help reducing such limits and increasing the diagnostic potential.

In this review, we will focus on circulating noncoding RNA molecules, including miRNAs, lncRNAs and circRNAs, discussing their potential value as non-invasive biomarkers for sarcoma diagnosis and prognosis prediction.

## 2. Circulating miRNAs Level May Function as Indicator of the Disease Status in Sarcoma Patients

This section addresses recent findings on a group of circulating miRNAs whose altered expression might be consistently associated with sarcoma progression, thus representing an important indicator of the clinical stage of disease, metastatic progression and/or therapeutic response.

First, miRNAs are single-stranded endogenous short noncoding RNAs that exert key regulatory functions by controlling the expression of specific target genes at post-transcriptional level, mainly by binding to the 3‘-untranslated regions of these mRNAs [12]. Growing evidence indicates that miRNAs may be differentially expressed in a tumor-specific fashion and may function either as oncogenes or tumor suppressors, depending on the cell type and tumor tissue [13]. Although deregulation in circulating miRNAs is often consistent with altered expression in tumor tissues, there is no unanimous consensus on whether miRNA alterations in tumor tissues parallel similar changes in the patients’ blood. In many tumors, miRNAs are secreted from cancer cells into the extracellular environment via multiple mechanisms [14], determining their presence not only in the surrounding cells and tissues, but also in circulating body fluids, such as serum, plasma and urine [15], where they remain highly stable. These interesting properties suggest their potential use as blood-based markers in cancer diagnosis and prognosis prediction [11,12].

**Figure 1:**
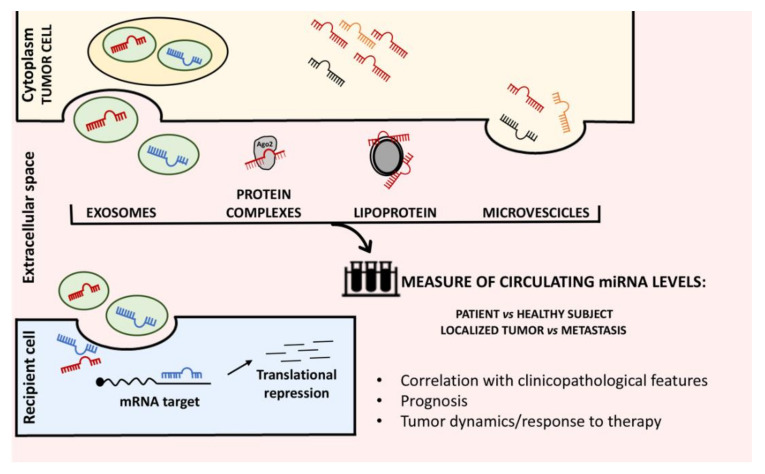
miRNAs release in the extracellular space. miRNAs can be released in the extracellular space through different ways, such as exosomes, by forming protein or lipoprotein complexes, and microvesicles. Once internalized in the recipient cell, miRNAs can act on the target mRNAs by achieving their translational repression. Circulating miRNA levels from blood samples showed differences between sarcoma patients and healthy subjects and between patients with localized tumor or metastatic disease. Circulating miRNA levels may correlate with the clinicopathological features of patients, with their prognosis and may change in response to therapeutic treatments.

Although in most cases, the mechanisms have not been elucidated yet, the correlation between changes in the circulating level of specific miRNAs and the tumorigenesis process (initiation, progression and metastasis formation) is well established [16]. To date, several studies have shown that circulating miRNAs may be released and transported through the exosome pathway, micro-vesicles or associated to protein complexes (Figure 1) [17]. Exosomes are a group of small extracellular vesicles (EVs) (30–100 nm in diameter) formed by the inward budding of endosomal membrane during the maturation of multivesicular endosomes and their subsequent fusion with the cell surface [18]. Exosomes can be secreted by multiple cell types and, due to their involvement in endocrine signals, are also present in many different body fluids, such as urine, synovial fluid, bile, cerebrospinal fluid, amniotic fluid, breast milk, blood and seminal plasma [18]. Depending on their origin and composition, exosomes can perform a variety of functions, in both physiological and pathological conditions. Tumor cells, as well as tumor microenvironments, in fact, can produce and secrete exosomes containing molecular cargos totally different from those produced by normal cells [19]. Such exosomes, called tumor-derived exosomes (TDEs) and tumor microenvironment exosomes (TMEs), respectively, exert key roles in the regulation of tumor growth, survival, and angiogenesis, as well as tumor invasion and metastasis, inducing epithelial-mesenchymal transition (EMT) and the formation of pre-metastatic niche [19,20]. Therefore, exosomes provide a novel way to spread effector messages between cells, including proteins, mRNAs and noncoding RNAs, such as miRNAs, lncRNAs and circRNAs [20].

### 2.1. Increased Circulating miRNAs Expression Associates with Worse Prognosis in Sarcoma Patients

It is well documented that miRNAs can serve oncogenic functions and that their abnormal expression represents a crucial step in the process of tumor initiation and progression. miRNAs, in fact, can regulate the expression of genes involved in the control of proliferation, angiogenesis and invasion [21,22]. Yuan and colleagues reported a correlation between the clinicopathological features of OS patients and high serum level of miR-21 [23], whose up-regulation was reported in several cancers [24,25]. Circulating miR-21 correlates with advanced Enneking stage, poor tumor response to neoadjuvant chemotherapy and reduced overall survival rate of patients, compared with healthy subjects [23]. Another study found that the expression level of miR-21 in the serum of OS patients before and after chemotherapy correlated with its expression in the corresponding tumor tissues [26]. Moreover, in patients with effective chemotherapy, miR-21 serum levels decreased after treatment, indicating that high miR-21 level may be associated to chemosensitivity of OS. In 2017, a global miRNA screening in serum of OS patients identified the miR-25-3p as prognostic factor for OS, whose increased concentration correlated with clinical features, including distant metastasis [27]. Importantly, the elevated serum level of miR-199a-5p was significantly reduced in OS patients after surgery [28], suggesting that the evaluation of miRNAs may be used to monitor tumor dynamics.

Other miRNAs whose increased serum level paralleled the expression in tumor tissue of OS patients, are miR-196a, miR-196b, miR-17, miR-221 and miR-300 [29,30,31,32,33]. The increased serum levels of miR-196a and miR-196b were associated with decreased overall survival and disease-free survival of OS patients. More interestingly, patients with high levels of these miRNAs in both serum and tumor tissues displayed worse prognosis, revealing the possibility for predicting the outcome of patients by analyzing miRNA concentration [29]. Similarly, the prognosis of patients with high serum expression of miR-17 [30] or miR-191 [31] was significantly worse in comparison with those exhibiting lower expression. miR-221 and miR-300 can be considered prognostic biomarkers: their elevated expression was associated with advanced clinical stage and positive metastasis status, correlating with poor outcome of OS patients [32,33]. Similarly, miRNA-421 was found upregulated not only in OS tumor tissue, compared with adjacent normal tissue, but also in the serum of OS patients, in comparison to healthy volunteers [34]. Furthermore, increased serum concentration of miR-9 [35], miR-27a [36] and miR-26a-5p [37] was found associated with advanced clinicopathological features, such as tumor stage and size, positive distant metastasis and correlated with a poor prognosis and shorter overall survival of OS patients. The expression levels of miR-29 family (miR-29a, miR-29b, and miR-29c) in OS tissues significantly correlated with those in the serum of patients. Interestingly, while the higher miR-29c level was not related to any clinicopathological features, high level of miR-29a and miR-29b was associated with high tumor grade, positive metastasis and recurrency [38]. In 2018, the circulating miR-215-5p, already proposed as hepatocellular carcinoma biomarker [39], was found upregulated in the serum of OS patients [40]. The elevated circulating level of another miRNA, miR-542-3p, was associated with advanced tumor stage and shorter free survival in OS patients [41].

As with OS, high serum levels of miRNAs were detected in other sarcomas. In 2017, miR-92b-3p was found increased in the serum of patients with SS compared with healthy subjects [42], suggesting the possibility that serum concentration of miR-92b-3p may be considered a non-invasive biomarker of SS. Importantly, circulating miR-375 is a good indicator of active Kaposi sarcoma (KS) in Acquired immunodeficiency syndrome (AIDS) patients [43]. In fact, its upregulated plasma expression decreased after chimeric antigen receptor (cART) T-cell-induced remission in most patients [43]. In RMS, the serum level of muscle-specific miRNAs, in particular miR-206, was significantly higher compared with healthy subjects [44].

Recently, Kohama and colleagues, by analyzing the expression profile of miRNAs in tissue and serum of dedifferentiated liposarcoma (DDLPS) patients, identified specific miRNAs highly expressed in both compartment (such as miR-1246, miR-4532 and miR-619-5p), suggesting their potential exploitation as biomarkers [45]. A microarray-based miRNA screening of blood RNA from DDLPS patients and healthy subjects identified miR-3613-3p as significantly upregulated [46]. Interestingly, miRNA-3613-3p showed higher level in patients with localized disease compared with patients with metastasis [46]. Similarly, high levels of miR-34a were found in the blood of ES patients with localized disease compared with metastatic patients [47]. Lastly, miR-1260b was found upregulated in patient with infiltrative myxofibrosarcoma (MFS) [48]. Results suggested that miRNA-1260b may be used as a biomarker for disease monitoring since its level correlated with tumor dynamics, as indicated by the decreased concentration in the blood of patients after tumor resection [48].

As mentioned above, mature miRNAs can be incorporated in exosomes. Although the precise mechanism of release into the extracellular environment remains largely unknown, numerous studies confirmed that several circulating miRNAs are enriched in exosomes [11]. In OS patients, enhanced serum exosomal miR-675 expression causes down-regulation of Calneuron 1 (*CALN1)*, thus influencing invasion and migration of cells [49]. Moreover, purified exosomes derived from metastatic OS cells increased the migration and invasion of fibroblasts, suggesting that exosomal miR-675 expression represents a biomarker for the metastatic process. Similarly, miR-15a was found highly expressed in exosomes of OS patients [50]. Thus, the hypothetical mechanism could involve exosomes internalization by OS cells and the control of cell cycle progression and tumor growth by miRNA-15a through repression of the *GATA2/MDM2* axis. Furthermore, miR-486-5p up-regulation was detected in serum-derived exosomes of patients with RMS [51], whose expression decreased after chemotherapy or cancer remission. This exosomal miRNA could be responsible of the enhanced tumorigenic phenotype of recipient cells.

By analyzing RNA isolated from LPS serum patients, Casadei and colleagues, showed that miR-25-3p and miR-92a-3p were secreted by LPS cells through exosomes [52], thus allowing the communication between tumor cells and the surrounding microenvironment. They acted by stimulating the secretion of pro-inflammatory IL-6 from tumor-associated macrophages, thus driving liposarcoma progression.

### 2.2. Down-Regulation of Specific Circulating miRNAs Correlates with Sarcoma Pathogenesis

miRNAs exert their functional regulation depending on the tumorigenic process which involves their target genes. As mentioned above, the level of circulating miRNAs may be related to their level in tumor tissue, as in the case of miRNA-34b, which decreased plasma level was observed in OS patients. In addition, plasma expression levels of miR-34b decreased in OS metastatic patients, compared with the non-metastatic ones [53]. miR-124 [54], miR-139-5p [55] and miR-101 [56] low serum concentration correlated with distant metastasis and worse OS patient survival. Interestingly, after chemotherapy, the serum level of miR-124 and miR-101 increased. A similar distribution between tumor tissue and serum was found also for the miR-375 [57], whereas low expression of miR-195 was associated with the clinicopathological features (advanced clinical stage and positive distant metastasis) of OS patients [58].

By analyzing control and disease-associated plasma from a genetically engineered mouse model of OS, some miRNAs were found differentially expressed [59]. Among them, miR-214 showed a similar behavior as in OS human samples: remarkably, its decreased plasma level in metastatic patients was associated with better prognosis [59]. Moreover, the survival time of OS patients with lower expression of miR-497 [60] was shorter than in patients with higher expression. In OS patients, the decreased serum level of miR-95-3p was associated with the progression and development of OS pathogenesis [61]. Notably, this miRNA was found upregulated in OS patients, compared with the healthy control [62]. miR-223, whose role was characterized in the myeloid lineage development [63], may act as tumor suppressor and its low expression in OS was associated with distant metastasis and clinical stage [64]. A key role for the miRNA-491 in OS lung metastasis and chemoresistance was also reported, and its decreased serum level correlated with increased metastasis, poor chemoresponse and low survival rate [65]. Similarly, lower miR-194 expression correlated with positive metastasis and advanced clinical stage in OS patients, even with no differences among the histological subtypes [66].

In 2012, miR-133b and miR-206 were found as the most downregulated miRNAs in OS tissue [67]. This downregulation in cancer tissues correlated with low serum level [68]. Remarkably, combined downregulation of serum and tissue miR-133b levels correlated with decreased overall survival and disease-free survival of patients, in comparison with single downregulation. Circulating miR-125b was found down-regulated in OS and ES patients [69,70]. Luo and colleagues observed that patients with unresectable OS showed lower miR-125b level, associated with advanced tumor stage [69].

In ES, low miR-125b expression was associated to poor response to chemotherapy [70]. Analysis of blood samples from RMS patients, revealed that miR-26a, miR-30b and -30c were reduced in comparison to healthy subjects [71]. No significant variation in the expression of these miRNAs was found between the two major RMS subtypes, alveolar rhabdomyosarcoma (ARMS) and embryonal rhabdomyosarcoma (ERMS). In addition, miR-26a correlated with enhanced risk of relapse and poor prognosis [71]. In uterine sarcoma, lower serum levels of miR-152 and miR-24 associated with worse prognosis [72].

Collectively, these studies suggest that circulating miRNAs might be useful as non-invasive biomarker in sarcoma (Table 1). Moreover, miRNA serum levels might be used for monitoring the therapeutic response and predicting sarcoma patients’ prognosis. However, these studies present some limitations, such as the small sample size and the fact that the detailed molecular mechanisms underlying the dysregulation of circulating miRNAs in sarcoma patients remain to be further elucidated.

## 3. Circulating lncRNAs as Novel Prognostic Factors for Sarcoma Malignancies

Long noncoding RNAs (lncRNAs) are, by definition, transcripts longer than 200 nt, expressed at lower levels than other RNA transcripts. Their expression can be tissue- and cell- specific, as well as dependent on epigenetic modification [73,74,75]. lncRNAs are characterized by low coding potential and rely on precise molecular interaction to fulfill their role in specific pathological contexts. Moreover, lncRNAs can serve as scaffolds in protein-protein or protein-nucleic acid interactions, thus contributing to the epigenetic changes and transcriptional events underlying regulation of gene expression signatures and miRNA stability. Specifically, these noncoding transcripts are able to modulate different cellular functions involved in tumorigenesis and several lncRNAs have been linked to oncogenic processes [76]. lncRNAs differentially expressed in normal or cancer tissues and metastases can be used as potential biomarkers for diagnosis, prognosis, and therapy [76]. Notably, some circulating lncRNAs are well suited for noninvasive analysis of patient samples, since they were found also in the serum, plasma, and other body fluids.

To date, only a limited number of circulating lncRNAs emerged as potential diagnostic or prognostic markers in sarcoma patients (Table 2) [77]. The Taurine Upregulated Gene 1 (*TUG1*) expression was found upregulated in OS patients [78,79]. *TUG1* is highly expressed in OS compared with adjacent healthy tissues, and its expression levels decreased in post-operative patients in comparison with pre-operative patients [79]. *TUG1* was also found in the plasma samples of OS patients and was associated with disease status [79]. Like *TUG1*, HNF1A Antisense RNA 1 (*HNF1A-AS1)* and Focally Amplified LncRNA on chromosome 1 (*FAL1*) were also monitored along with disease progression, and their expression was found increased with relapse [80].

High expression of *HNF1A-AS1* conferred poor survival rate to patients [80]. Remarkably, *HNF1A-AS1* expression was higher in the serum of pre-operative patients compared with healthy donors or post-operative patients [80]. Serum samples of OS patients and healthy controls were analyzed also for expression levels of *FAL1*. Results showed that *FAL1* expression decreased in post-operative patients and in healthy controls. Interestingly, serum *FAL1* expression was significantly higher in patients displaying benign bone lesions or after chemotherapy [81].

The expression level of other lncRNAs was correlated with survival probability in OS. Like the cases already discussed, high levels of *EPEL*, *MALAT1*, *FGD5-AS1* and *LINC01278* were associated with poor prognosis [82,83]. Particularly, the expression of *EPEL* (E2F-mediated cell proliferation enhancing lncRNA) was detected in the serum of OS patients, and was found upregulated in patients with distant metastases with respect to the control group, and non-distant metastasis [82]. Over the years, several studies highlighted the lncRNA *MALAT1* as a diagnostic biomarker for multiple malignancies, carcinomas, non-small-cell lung cancer, and epithelial ovarian cancer [84]. In OS, high expression of *MALAT1* was associated with poor overall and progressive-free survival [83]. Likewise, the lncRNA *FGD5-AS1* was found abnormally highly expressed in many cancer tissues and associated with poor prognosis [85]. Of note, it was also found highly expressed in the serum of OS patients [74].

Although great efforts were devoted to select reliable circulating prognostic factors in sarcoma, the molecular mechanisms driving the production of these circulating lncRNAs are still poorly characterized, and little is known about the function of these circulating lncRNAs in sarcoma tumors. Some investigations were able to define the export mechanism. In particular, for lncRNAs predominantly localized in the cytoplasm compartment (cc-lncRNAs) it was reported that the export mechanism was similar to miRNA molecules, based on membrane-bound vesicles or through a vesicle-free RNA-binding protein dependent pathway, in a miRNAs similar fashion [86,87,88]. By contrast, some nuclear lncRNAs could be released from cancer cells upon death, as for *ATB*, *HNF1A-AS1*, *LINC01278*, *LINC01354*, *LINK-A* and *UCA1* [89,90,91,92,93]. The Activated by TGF-β lncRNA (*lncRNA-ATB*) was investigated as a potential novel non-invasive biomarker for OS: in fact, high *lncRNA-ATB* levels showed worse recurrence-free and overall survival [89]. *LINC01278* is highly expressed in OS, and patients with high *LINC01278* expression showed poor prognosis [94], like *UCA1* [90,91]. Moreover, the analysis of blood samples from chondrosarcoma patients and healthy volunteers revealed that the expression level of RAMP2 Antisense RNA 1 *(RAMP2-AS1)* was higher in the pathological condition. The level of *RAMP2-AS1* varied along with the stage of the disease, with higher expression in the T2 stage and in patients with distant metastasis (M1 stage) [95]. Kaplan-Meier analysis revealed that high *RAMP2-AS1* levels are associated with poor overall survival [95].

Notably, the lncRNAs mentioned above were all found upregulated in patients’ biopsies. By contrast, serum levels of the lncRNA Heart and Neural crest Derivatives expressed 2-antisense RNA 1 (lncRNA *HAND2-AS1*) were significantly higher in control subjects compared with OS patients [96]. Similarly, plasma levels of lncRNA-*NEF* significantly decreased in patients with OS [97]. ROC curve analysis indicated their potential diagnostic value in OS. Interestingly, *NEF* downregulation significantly correlated with the increase of miRNA-21 in plasma samples [97].

Although the feasibility of using circulating lncRNAs as putative cancer biomarkers has been extensively reported, many aspects can interfere with the quantification of circulating lncRNAs. Thus, standardization of extraction procedures and quantification techniques need to be deeply investigated and ameliorated.

Notably, lncRNA traits are specific to cancer types and features, such as oncogene expression, hormone responsiveness and drug treatment. This last aspect could suggest the potential use of circulating lncRNAs as pharmacodynamic markers.

**Table 2:** List of lncRNAs identified in sarcoma.

LncRNA Name	Expression Pattern	Sarcoma Type/ No. of Patients	Similar Expression in Tumor Tissue	Disease Monitoring	Circulating Expression also in Other Cancers
*TUG1*	Upregulated (plasma)	OS [78,79]/ 40,76	yes	Decreased expression in post-operative patients Increased level in case of relapse	LAD [98] Breast cancer [99] MM [100]
*HNF1A-AS1*	Upregulated (serum)	OS [80]/72	yes	Decreased expression in patients with post-operative chemotherapy Increased level in case of relapse	ESCC [101]
*FAL1*	Upregulated (serum)	OS [81]/39	yes	Decreased expression in post-operative patients	GC [102] HCC [103]
*EPEL*	Upregulated (serum)	OS [82]/39	yes	Not analyzed	No data available
*MALAT1*	Upregulated (serum)	OS [83]/46	yes	Not analyzed	Breast cancer [104] MM [105] gastric adenocarcinoma [106] NSLC [107] glioblastoma multiforme [108] EOC [109]
*FGD5-AS1*	Upregulated (serum)	OS [85]/97	yes	Not analyzed	Thyroid Cancer [110]
*ATB*	Upregulated (serum)	OS [89]/60	yes	Increased expression in case of relapse	CRC [111] HCC [112] Breast cancer [113]
*LINC01278*	Upregulated (serum)	OS [94]/66	yes	Not analyzed	No data available
*RAMP2-AS1*	Upregulated (serum)	Chondrosarcoma [95]/45	Not reported	Not analyzed	No data available
*HAND2* *-* *AS1*	Downregulated (serum)	OS [96]/48	yes	not analyzed	No data available
*NEF*	Downregulated (plasma)	OS [97]/49	Not reported	Not analyzed	NSCLC [114] IHCC [115] GC [116] Glioma [117]
*LINC01354*	Upregulated (serum)	OS [93]/30	yes	Not analyzed	No data available
*UCA1*	Upregulated (serum)	OS [90,91]/85	yes	Not analyzed	Bladder cancer [118] HCC [119] PC [120] CRC [121,122]
*LINK-A*	Upregulated (plasma of metastatic patients)	OS [92]/62	Not reported	Not analyzed	OC [123]

LAD: Lung adenocarcinoma; MM: Multiple myeloma; ESCC: Esophageal squamous cell carcinoma; GC: Gastric cancer; HCC: Human hepatocarcinoma; NSCLC: Non-small-cell lung carcinoma; EOC: Epithelial ovarian cancer; CRC: Colorectal cancer; IHCC: Intrahepatic cholangiocarcinoma; PC: Prostate cancer; OC: Ovarian cancer. Quantification of circulating lncRNAs levels was performed by RT-qPCR in all identified studies.

## 4. Circulating circRNAs in Sarcoma Diagnosis and Prognosis

Circular RNAs (circRNAs) represent an abundant class of covalently closed and mostly noncoding RNA molecules [124]. Most of them are produced from protein-coding genes through a particular splicing mechanism, called back-splicing, that, by exploiting the canonical spliceosome machinery, allows joining of a downstream donor (5′ splice site) and an upstream acceptor (3′ splice site) [124,125]. Despite back-splicing is less efficient than canonical linear counterpart [126], the presence of inverted *Alu* repeats, non-repetitive complementary sequences, and binding sites for dimerizing RNA-binding proteins (RBPs), can favor the formation of the looping structure [127,128].

CircRNAs are involved at different levels in the regulation of cellular homeostasis and are characterized by tissue-specific expression patterns [129,130] and high stability [131]. They execute their function through different mechanisms. First, they can act as miRNA sponges, preventing the interaction between specific miRNAs and their mRNA targets [132]. In addition, circRNAs interact with different RBPs, thus acting as scaffolds in the formation of multiprotein complexes. The resulting structures work as protein sponges, able to inhibit specific protein functions, protein translation or even able to recruit proteins to specific subcellular compartments and organelles [133,134,135]. Despite the lack of 5′cap and poly(A) tail, some circRNAs can act as protein templates, thanks to the presence in their sequences of internal ribosome entry sites (IRES) or m6a RNA modifications, that allow a cap-independent translation [136,137,138]. All together, these characteristics help explaining the recent emerging roles of circRNAs in cancer [139,140].

Depending on their sequence and cellular localization, circRNAs can be involved in the regulation of gene expression profile of tumor cells, participating, as oncogenes or tumor suppressors, at different levels in tumor onset and progression. As already reported for miRNAs and lncRNAs, circRNAs are also present in body fluids, such as plasma, serum, saliva and urine [11], showing different expression patterns in cancer patients and healthy subjects [141]. This observation makes circRNAs a promising tool as non-invasive biomarkers in tumor diagnosis and prognosis, as well as novel potential therapeutic targets.

### 4.1. Free Circulating circRNAs in Liquid Biopsies

Recent studies are bringing to light the role of circRNAs as novel biomarkers also in sarcomas, particularly in OS [142,143].

Notably, a recent study identified hsa_circ_0081001 as highly expressed in the serum of OS patients compared with healthy controls; remarkably, hsa_circ_0081001 positively correlated with chemoresistance, lung metastasis and tumor recurrence [142], making hsa_circ_0081001 a new potential diagnostic and prognostic biomarker. A similar study identified hsa_circ_0000885 in the serum of OS patients, mostly in patients with Enneking stage IIB and III OS, compared with tumors at earlier stages or healthy controls. Also in this case, a positive correlation was observed between high serum levels of the hsa_circ_0000885 and low rates of disease-free survival, chemoresistance and lung metastasis. Conversely, after chemotherapy and surgery, the level of hsa_circ_0000885 dramatically decreased, highlighting its possible eligibility as diagnostic and prognostic biomarker in OS [143].

Although these studies underline the strong potential of circRNAs in body fluids as new key tools in cancer diagnosis and prognosis, to date their clinical applicability is still limited. First, the lack of standard nomenclature systems makes difficult to recognize the same circRNA in different studies, hindering clinical reproducibility. Second, the results obtained in the studies described above are obtained by case-control analyses on small samples with evident phenotypes. Additional studies on a larger number of patients with different clinical characteristics are needed to evaluate the sensitivity and specificity of circRNAs in diagnosis, necessary to translate them into clinical practice. Finally, another obstacle is represented by the low abundance of circRNAs in body fluids, which makes their detection very hard [144].

### 4.2. Exosomal circRNAs

Blood exosomes are particularly enriched in circRNAs [145]. Among other RNA molecules, circRNAs are attracting particular attention, due to their stability and regulatory roles in gene expression. These characteristics indicate that the exosomal circRNA content could be not only a passive reflection of the cellular content, but, instead, the consequence of particularly active sorting machineries.

Recent studies highlighted the potential role of exosomal circRNAs as prognostic and diagnostic biomarkers in cancer, identifying a close correlation between circRNAs carried by exosomes and OS progression. High levels of hsa_circ_103801 were found in OS patients’ serum exosomes, showing a negative correlation with patient survival [146]. Analysis of the human OS cell line MG63 showed higher expression level of hsa_circ_103801 in exosomes of cisplatin (CDDP)-resistant compared with sensitive MG63 cells. Moreover, transfer of exosomes containing hsa_circ_103801 from CDDP-resistant to CDDP-sensitive cells, allowed acquisition of resistance to the chemotherapeutic agent by inducing the expression of resistance-related proteins. Remarkably, Li and colleagues observed lower expression levels of hsa_circ_0000190 in OS cell lines and tissues compared with healthy controls, showing a negative correlation between its expression pattern and the ability of tumor cells to proliferate, migrate and invade other tissues [147]. By performing molecular analysis in OS cell lines, the authors found that hsa_circ_0000190 acts as a miRNA sponge, interacting with miR-767-5p and preventing its interaction with the mRNA encoding the tumor suppressor TET1 [147]. Furthermore, low expression of hsa_circ_0000190 was observed in the EVs from the plasma of OS patients, underlying a potential role as diagnostic biomarker and therapeutic agent [147].

Many advantages are provided by the application of exosomal circRNAs as tumor biomarkers. As first, exosomes derived from tumor cells carry disease-specific circRNAs in the peripheral blood, allowing to easily discriminate these RNA molecules from circulating circRNAs produced by untransformed cells. Next, the size of exosomes and their molecular composition, reflects the specific type and pathophysiological state of the producing cells [148]. As last, exosomes protect RNA molecules from degradation by RNases in the blood [149].

In summary, current knowledge makes clear how research on circulating circRNAs in sarcomas is still in its infancy. More focused studies are needed to improve their understanding and exploitation. As first, it would be necessary to understand in more detail the circRNA expression pattern in sarcoma patients and their mechanisms of action. Next, it would be important to improve technologies aimed at their detection, as well as the identification of a unique nomenclature to render easier the study of these circRNAs in different tumor types. All together, these studies will allow to open the way towards clinical application of new RNA-based molecular tools for cancer diagnosis, prognosis and therapy.

## 5. Methods and Technological Challenges in the Early Detection of Circulating RNAs

Given the relevant alterations of circulating RNAs in cancer patients, paralleling disease progression, therapy treatment and tumor recurrence, the fundamental need has emerged to improve extraction and amplification procedures and to standardize detection techniques. High-throughput techniques and next-generation sequencing technologies are greatly helping to achieve this goal, also enabling the discovery of novel tumor-associated ncRNAs.

In this paragraph we will describe general techniques used to detect circulating RNAs, listed in Table 3. Several detection methods are available for rapid quantification, with good sensitivity and specificity; however, more appropriate strategies depend on the specific class of RNA to measure; miRNAs levels can be easily measured by real-time quantitative PCR (RT-qPCR), and microarray-based technologies. Microarray technologies showed high sensitivity, but they allow for identifying only known RNAs [150], whereas novel cancer-specific variants could not be detected. Droplet digital PCR allows for analyzing samples of small quantity, without laborious preparative procedures. This technique requires optimized enzymes, probes and standardized controls. To date, next-generation sequencing represents the most favorable approach to detecting both known and unknown RNA molecules, although the required quantity of samples might be limiting. The last frontier in detecting circRNAs are isoCirc and CIRI-long technologies, representing an evolution of third-generation sequencing optimized to minimize low-accuracy defects [151,152]. isoCirc combines rolling circle amplification and nanopore long-read sequencing to characterize full-length circRNA isoforms. After total RNAs extraction from a biological sample, circRNAs can be enriched and linear RNAs depleted through ribosomal RNA (rRNA) removal and RNase R treatment. Next, tandem repeats can be detected from long reads and used to generate consensus sequences. For each read, a concatemer of two copies of the consensus sequence is mapped to the genome to identify the back-splice junction and forward-splice junctions within the circRNA. In this way, isoCirc enables identification of high-confidence back-splice junctions and full-length circRNA isoforms (https://github.com/Xinglab/isoCirc, 27 July 2022) [151]. To overcome difficulties in reconstructing the sequence of circRNAs from short RNA sequencing reads, a new approach was developed, coupling the nanopore technology with a new algorithm called circRNA identifier using long-read sequencing data (CIRI-long) [152] (https://ciri-cookbook.readthedocs.io/en/latest/CIRI-long_sequencing.html, 27 July 2022). This method takes advantage of nanopore long reads and enables unbiased reconstruction of full-length circRNA sequences [152].

Another major issue in circulating RNA detection is the normalization of data. Internal control helps to ensure data quality and reduce variation among sample. Housekeeping genes are extensively used for normalization, although they do not represent very well the circulating class of RNA molecules. Another limit of the described strategies is that they are not able to discriminate RNA isolated from exosomes produced by tumor from those by untransformed cells. Cationic liposome nanoparticle biochips represent a new strategy that can overcome this issue [153]. Nanoparticles containing molecular beacons tethered on the surface of a gold-coated glass can trap the negative charged exosomes and recognize specific surface substrates marking different types of exosomes. The main limit of this method is the ability to perform a qualitative analysis of circRNA expression levels in extracellular vesicles but not the quantitative analysis needed to understand the real amounts of these circRNAs in exosomes.

In short, all stages of sample collection, preparation and RNA detection require standardized procedures for performing reliable prognostic and diagnostic analysis. Interindividual variability and tumor type also require consideration since race, gender and age, together with genetic polymorphisms, could impact the level of circulating RNAs [154,155].

## 6. Conclusions

Circulating RNA molecules display great potential as cancer biomarkers and are critically important for understanding disease-associated physio-pathological features and cancer dynamics, opening opportunities in therapeutic targeting. In addition to the valuable non-invasive nature of blood, urine and saliva sampling, liquid biopsies also allow for serial sample collection at different time points relative to treatments or stage of disease. These intrinsic properties guarantee an overall estimation of tumor characteristics, supporting treatment decisions and allowing to monitor stepwise response to therapy.

Remarkably, circulating noncoding RNAs not only regulate the expression of genes that control fundamental biological functions but also mediate cell-to-cell communication, thus impacting gene expression in recipient cells and influencing the tumor microenvironment. Thus, they act similarly to soluble molecular messengers, such as cytokines, chemokines and hormones, representing key molecules in the balance of the equilibrium of tissue homeostasis and malignant transformation.

The literature reviewed herein supports the potential use of circulating noncoding RNAs, in particular miRNAs, as both diagnostic and prognostic biomarkers. However, despite encouraging results in the assessment of circulating RNAs in body fluids, the origin and function of these RNAs in the extracellular environment remains poorly understood. Moreover, studies with large cohorts of patients are absolutely required to validate their use as reproducible diagnostic biomarkers. Lastly, technical advances to improve standardization in sample preparation, quality assessment and quantification are urgently needed to guarantee their adoption into clinical practice. This possibility opens the path to new perspectives in the development of non-invasive markers for sarcoma pathogenesis.

## Figures and Tables

**Table 1:** List of miRNAs identified in sarcoma body fluids.

miRNA Name	Expression Pattern	No. of Patients	Similar Expression Pattern in Tumor Tissue	Disease Monitoring	Method to Identify/ Quantify miRNA
Osteosarcoma					
miR-21 [23,26]	Upregulated (serum)	65/69	Not reported/yes	Not analyzed	RT-qPCR
miR-25-3p [27]	Upregulated (serum)	10	yes	Decrease after tumor resection or during chemotherapy	Microarray/ RT-qPCR
miR-196a/b [29]	Upregulated (serum)	100	yes	Not analyzed	RT-qPCR
miR-17 [30]	Upregulated (serum)	46	yes	Not analyzed	RT-qPCR
miR-191 [31]	Upregulated (serum)	100	yes	Not analyzed	RT-qPCR
miR-221 [32]	Upregulated (serum)	108	yes	Not analyzed	RT-qPCR
miR-300 [33]	Upregulated (serum)	114	yes	Decreased in patients with curative surgeries	RT-qPCR
miR-421 [34]	Upregulated (serum)	40	yes	Not analyzed	RT-qPCR
miR-9 [35]	Upregulated (serum)	118	Not reported	Not analyzed	RT-qPCR
miR-27a [36]	Upregulated (serum)	166	Not reported	Not analyzed	RT-qPCR
miR-26a-5p [37]	Upregulated (serum)	243	yes	Not analyzed	RT-qPCR
miR-29 family [38]	Upregulated (serum)	80	yes	Not analyzed	RT-qPCR
miR-215-5p [40]	Upregulated (serum)	15	Not reported	Not analyzed	miRNA Low Density Arrays (TLDAs)/ RT-qPCR
miR-542-3p [41]	Upregulated (serum)	76	Not reported	Not analyzed	RT-qPCR
miR-675 [49]	Upregulated (serum exosomes)	10	Not reported	Not analyzed	RNA sequencing/ RT-qPCR
miR-15a [50]	Upregulated (serum exosomes)	31	no	Not analyzed	RT-qPCR
miR-34b [53]	Downregulated (plasma)	133	yes	Not analyzed	RT-qPCR
miR-124 [54]	Downregulated (serum)	114	yes	Increased after chemotherapy treatment	RT-qPCR
miR-139-5p [55]	Downregulated (serum)	98	Not reported	Not analyzed	RT-qPCR
miR-101 [56]	Downregulated (serum)	152	Not reported	Increased after chemotherapy treatment	RT-qPCR
miR-375 [57]	Downregulated (serum)	95	Not reported	Not analyzed	RT-qPCR
miR-195 [58]	Downregulated (serum)	195	Not reported	Not analyzed	RT-qPCR
miR-214 [59]	Downregulated (plasma of metastatic patients)	40	Not reported	Not analyzed	RT-qPCR
miR-497 [60]	Downregulated (serum)	185	Not reported	Not analyzed	RT-qPCR
miR-223 [64]	Downregulated (serum)	112	Not reported	Not analyzed	RT-qPCR
miR-491 [65]	Downregulated (serum)	102	Not reported	Not analyzed	RT-qPCR
miR-194 [66]	Downregulated (serum)	124	Not reported	Increased after surgery	RT-qPCR
miR-133b [67]	Downregulated (serum)	100	yes	Not analyzed	RT-qPCR
miR-206 [68]	Downregulated (serum)	100	yes	Not analyzed	RT-qPCR
miR-125b [69]	Downregulated (plasma of unresectable OS patients)	138	Not reported	Not analyzed	RT-qPCR
Synovial sarcoma					
miR-92b-3p [42]	Upregulated (serum)	12	Not reported	Decreased after tumour resection and adjuvant chemotherapy	Microarray/ RT-qPCR
Kaposi’s sarcoma					
miR-375 [43]	Upregulated (serum)	10	Not reported	Decreased after cART-cell induced remission	Microarray/ RT-qPCR
Dedifferentiated liposarcoma					
miR-1246 [45]	Upregulated (serum)	17	yes	Not analyzed	Microarray/ RT-qPCR
miR-4532 [45]	Upregulated (serum)	17	yes	Not analyzed	Microarray/ RT-qPCR
miR-619-5p [45]	Upregulated (serum)	17	yes	Not analyzed	Microarray/ RT-qPCR
miR-3613-3p [46]	Upregulated (serum)	6	Not reported	Not analyzed	Affymetrix GeneChip Array/RT-qPCR
Rhabdomyosarcoma					
miR-206 [44]	Upregulated (serum)	10	yes	Decreased after treatment	RT-qPCR
miR-26a [71]	Downregulated (plasma)	30	Not reported	Not analyzed	ddPCR
miR-486-5p [51]	Upregulated (serum exosome)	10	Not reported	Decreased after chemotherapy	Microarray/ RT-qPCR
Ewing sarcoma					
miR-34a [47]	Upregulated (plasma of localized disease patient)	31	Not reported	Increased after chemotherapy	RT-qPCR
miR-125b [70]	Downregulated (serum)	63	Not reported	Decreased in case of poor response to chemotherapy	Microarray/ RT-qPCR
Liposarcoma					
miR-25-3p [52]	Upregulated (plasma vesicles)	16	no	Not analyzed	NanoString nCounter miRNA expression assay/ RT-qPCR
miR-92a-3p [52]	Upregulated (plasma vesicles)	16	no	Not analyzed	RT-qPCR
Myxofibrosarcoma					
miR-1260b [48]	Upregulated (serum)	5	no	Decreased postoperatively	Microarray/ RT-qPCR
Uterine sarcoma					
miR-24 [72]	Downregulated (serum)	101	Not reported	Not analyzed	RT-qPCR
miR-152 [72]	Downregulated (serum)	101	Not reported	Not analyzed	RT-qPCR

RT-qPCR: reverse transcription quantitative real-time PCR; ddPCR: droplet digital PCR; TLDAs: TaqMan low-density arrays.

**Table 3:** List of methodological procedures for detecting circulating noncoding RNAs.

Circulating RNAs Detection Techniques				
Technique	Sensitivity	Specificity	Advantages	Disadvantages
RT-qPCR	low	Dependent to primer design	Quick and easy to perform	Analysis is limited to a restricted number of targets, normalization relative to housekeeping genes
Microarray	medium	Dependent to probe design and density	Quick and easy to perform	Probes can limit the detection of novel mutations
Droplet digital PCR	high	good	Absolute quantification, ideal for low input target concentration	Limited number of targets, prior knowledge of mutations is required
Next generation sequencing	high	for detection of genetic and epigenetic changes	Simultaneous profiling of several genes	Specialized bioinformatic approach
isoCirc	very high (for circRNAs)	reduced low-accuracy defects	70-fold enrichment of circRNA reads compared with RNase R-treated short-read libraries High reproducibility among technical replicates	Specialized bioinformatic approach
CIRI-long	very high (for circRNAs)	reduced low-accuracy defects	mitochondrial circRNAs detection	Specialized bioinformatic approach
Cationic liposome nanoparticle biochips	very high	to distinguish cancer cell-derived exosomal miRNAs from normal cell-derived exosomal miRNAs	detection of tumor-derived exosomal microRNAs with high specificity and sensitivity	Specialized technical procedures and instruments

## References

[1] Wunder J.S., Nielsen T.O., Maki R.G., O’Sullivan B., Alman B.A. (2007). Opportunities for improving the therapeutic ratio for patients with sarcoma. Lancet Oncol..

[2] Grünewald T.G., Alonso M., Avnet S., Banito A., Burdach S., Cidre-Aranaz F., Di Pompo G., Distel M., Dorado-Garcia H., Garcia-Castro J. (2020). Sarcoma treatment in the era of molecular medicine. EMBO Mol. Med..

[3] Zöllner S.K., Amatruda J.F., Bauer S., Collaud S., de Álava E., DuBois S.G., Hardes J., Hartmann W., Kovar H., Metzler M. (2021). Ewing Sarcoma-Diagnosis, Treatment, Clinical Challenges and Future Perspectives. J. Clin. Med..

[4] Dancsok A.R., Asleh-Aburaya K., Nielsen T.O. (2017). Advances in sarcoma diagnostics and treatment. Oncotarget.

[5] Ignatiadis M., Sledge G.W., Jeffrey S.S. (2021). Liquid biopsy enters the clinic—Implementation issues and future challenges. Nat. Rev. Clin. Oncol..

[6] Leary R.J., Sausen M., Kinde I., Papadopoulos N., Carpten J.D., Craig D., O’Shaughnessy J., Kinzler K.W., Parmigiani G., Vogelstein B. (2012). Detection of chromosomal alterations in the circulation of cancer patients with whole-genome sequencing. Sci. Transl. Med..

[7] Wan J.C.M., Massie C., Garcia-Corbacho J., Mouliere F., Brenton J.D., Caldas C., Pacey S., Baird R., Rosenfeld N. (2017). Liquid biopsies come of age: Towards implementation of circulating tumour DNA. Nat. Rev. Cancer.

[8] Namløs H.M., Boye K., Meza-Zepeda L.A. (2020). Cell-free DNA in blood as a noninvasive insight into the sarcoma genome. Mol. Asp. Med..

[9] Klega K., Imamovic-Tuco A., Ha G., Clapp A.N., Meyer S., Ward A., Clinton C., Nag A., Van Allen E., Mullen E. (2018). Detection of Somatic Structural Variants Enables Quantification and Characterization of Circulating Tumor DNA in Children With Solid Tumors. JCO Precis. Oncol..

[10] Shukla N.N., Patel J.A., Magnan H., Zehir A., You D., Tang J., Meng F., Samoila A., Slotkin E.K., Ambati S.R. (2017). Plasma DNA-based molecular diagnosis, prognostication, and monitoring of patients with. JCO Precis. Oncol..

[11] Anfossi S., Babayan A., Pantel K., Calin G.A. (2018). Clinical utility of circulating non-coding RNAs—An update. Nat. Rev. Clin. Oncol..

[12] Valihrach L., Androvic P., Kubista M. (2020). Circulating miRNA analysis for cancer diagnostics and therapy. Mol. Asp. Med..

[13] Peng Y., Croce C.M. (2016). The role of MicroRNAs in human cancer. Signal Transduct. Target. Ther..

[14] Cui M., Wang H., Yao X., Zhang D., Xie Y., Cui R., Zhang X. (2019). Circulating MicroRNAs in Cancer: Potential and Challenge. Front. Genet..

[15] Cortez M.A., Bueso-Ramos C., Ferdin J., Lopez-Berestein G., Sood A.K., Calin G.A. (2011). MicroRNAs in body fluids—The mix of hormones and biomarkers. Nat. Rev. Clin. Oncol..

[16] Wang H., Peng R., Wang J., Qin Z., Xue L. (2018). Circulating microRNAs as potential cancer biomarkers: The advantage and disadvantage. Clin. Epigenetics.

[17] Sohel M.M.H. (2020). Circulating microRNAs as biomarkers in cancer diagnosis. Life Sci..

[18] Mashouri L., Yousefi H., Aref A.R., Ahadi A.M., Molaei F., Alahari S.K. (2019). Exosomes: Composition, biogenesis, and mechanisms in cancer metastasis and drug resistance. Mol. Cancer.

[19] De Toro J., Herschlik L., Waldner C., Mongini C. (2015). Emerging roles of exosomes in normal and pathological conditions: New insights for diagnosis and therapeutic applications. Front. Immunol..

[20] Quail D.F., Joyce J.A. (2013). Microenvironmental regulation of tumor progression and metastasis. Nat. Med..

[21] Baranwal S., Alahari S.K. (2010). miRNA control of tumor cell invasion and metastasis. Int. J. Cancer.

[22] Frixa T., Donzelli S., Blandino G. (2015). Oncogenic MicroRNAs: Key Players in Malignant Transformation. Cancers.

[23] Yuan J., Chen L., Chen X., Sun W., Zhou X. (2012). Identification of serum microRNA-21 as a biomarker for chemosensitivity and prognosis in human osteosarcoma. J. Int. Med. Res..

[24] Fulci V., Chiaretti S., Goldoni M., Azzalin G., Carucci N., Tavolaro S., Castellano L., Magrelli A., Citarella F., Messina M. (2007). Quantitative technologies establish a novel microRNA profile of chronic lymphocytic leukemia. Blood.

[25] Feng Y.H., Wu C.L., Tsao C.J., Chang J.G., Lu P.J., Yeh K.T., Uen Y.H., Lee J.C., Shiau A.L. (2011). Deregulated expression of sprouty2 and microRNA-21 in human colon cancer: Correlation with the clinical stage of the disease. Cancer Biol. Ther..

[26] Hua Y., Jin Z., Zhou F., Zhang Y.Q., Zhuang Y. (2017). The expression significance of serum MiR-21 in patients with osteosarcoma and its relationship with chemosensitivity. Eur. Rev. Med. Pharmacol. Sci..

[27] Fujiwara T., Uotani K., Yoshida A., Morita T., Nezu Y., Kobayashi E., Uehara T., Omori T., Sugiu K., Komatsubara T. (2017). Clinical significance of circulating miR-25-3p as a novel diagnostic and prognostic biomarker in osteosarcoma. Oncotarget.

[28] Zhou G., Lu M., Chen J., Li C., Zhang J., Shi X., Wu S. (2015). Identification of miR-199a-5p in serum as noninvasive biomarkers for detecting and monitoring osteosarcoma. Tumor Biol..

[29] Zhang C., Yao C., Li H., Wang G., He X. (2014). Combined elevation of microRNA-196a and microRNA-196b in sera predicts unfavorable prognosis in patients with osteosarcomas. Int. J. Mol. Sci..

[30] Li S., Gao Y., Wang Y., Wang K., Dai Z.P., Xu D., Liu W., Li Z.L., Zhang Z.D., Yang S.H. (2016). Serum microRNA-17 functions as a prognostic biomarker in osteosarcoma. Oncol. Lett..

[31] Wang T., Ji F., Dai Z., Xie Y., Yuan D. (2015). Increased expression of microRNA-191 as a potential serum biomarker for diagnosis and prognosis in human osteosarcoma. Cancer Biomark..

[32] Yang Z., Zhang Y., Zhang X., Zhang M., Liu H., Zhang S., Qi B., Sun X. (2015). Serum microRNA-221 functions as a potential diagnostic and prognostic marker for patients with osteosarcoma. Biomed. Pharm..

[33] Liu J.D., Xin Q., Tao C.S., Sun P.F., Xu P., Wu B., Qu L., Li S.Z. (2016). Serum miR-300 as a diagnostic and prognostic biomarker in osteosarcoma. Oncol. Lett..

[34] Zhou S., Wang B., Hu J., Zhou Y., Jiang M., Wu M., Qin L., Yang X. (2016). miR-421 is a diagnostic and prognostic marker in patients with osteosarcoma. Tumor Biol..

[35] Fei D., Li Y., Zhao D., Zhao K., Dai L., Gao Z. (2014). Serum miR-9 as a prognostic biomarker in patients with osteosarcoma. J. Int. Med. Res..

[36] Tang J., Zhao H., Cai H., Wu H. (2015). Diagnostic and prognostic potentials of microRNA-27a in osteosarcoma. Biomed. Pharm..

[37] Xie X.Y., Chen X.M., Shi L., Liu J.W. (2021). Increased expression of microRNA-26a-5p predicted a poor survival outcome in osteosarcoma patients: An observational study. Medicine.

[38] Hong Q., Fang J., Pang Y., Zheng J. (2014). Prognostic value of the microRNA-29 family in patients with primary osteosarcomas. Med. Oncol..

[39] Zhang Z.Q., Meng H., Wang N., Liang L.N., Liu L.N., Lu S.M., Luan Y. (2014). Serum microRNA 143 and microRNA 215 as potential biomarkers for the diagnosis of chronic hepatitis and hepatocellular carcinoma. Diagn. Pathol..

[40] Monterde-Cruz L., Ramírez-Salazar E.G., Rico-Martínez G., Linares-González L.M., Guzmán-González R., Delgado-Cedillo E., Estrada-Villaseñor E., Valdés-Flores M., Velázquez-Cruz R., Hidalgo-Bravo A. (2018). Circulating miR-215-5p and miR-642a-5p as potential biomarker for diagnosis of osteosarcoma in Mexican population. Hum. Cell.

[41] Li Q., Song S., Ni G., Li Y., Wang X. (2018). Serum miR-542-3p as a prognostic biomarker in osteosarcoma. Cancer Biomark..

[42] Uotani K., Fujiwara T., Yoshida A., Iwata S., Morita T., Kiyono M., Yokoo S., Kunisada T., Takeda K., Hasei J. (2017). Circulating MicroRNA-92b-3p as a Novel Biomarker for Monitoring of Synovial Sarcoma. Sci. Rep..

[43] Piano M.A., Gianesello L., Grassi A., Del Bianco P., Mattiolo A., Cattelan A.M., Sasset L., Zanovello P., Calabrò M.L. (2019). Circulating miRNA-375 as a potential novel biomarker for active Kaposi’s sarcoma in AIDS patients. J. Cell. Mol. Med..

[44] Miyachi M., Tsuchiya K., Yoshida H., Yagyu S., Kikuchi K., Misawa A., Iehara T., Hosoi H. (2010). Circulating muscle-specific microRNA, miR-206, as a potential diagnostic marker for rhabdomyosarcoma. Biochem. Biophys. Res. Commun..

[45] Kohama I., Asano N., Matsuzaki J., Yamamoto Y., Yamamoto T., Takahashi R.U., Kobayashi E., Takizawa S., Sakamoto H., Kato K. (2021). Comprehensive serum and tissue microRNA profiling in dedifferentiated liposarcoma. Oncol. Lett..

[46] Fricke A., Cimniak A.F.V., Ullrich P.V., Becherer C., Bickert C., Pfeifer D., Heinz J., Stark G.B., Bannasch H., Braig D. (2018). Whole blood miRNA expression analysis reveals miR-3613-3p as a potential biomarker for dedifferentiated liposarcoma. Cancer Biomark..

[47] Sciandra M., De Feo A., Parra A., Landuzzi L., Lollini P.L., Manara M.C., Mattia G., Pontecorvi G., Baricordi C., Guerzoni C. (2020). Circulating miR34a levels as a potential biomarker in the follow-up of Ewing sarcoma. J. Cell Commun. Signal..

[48] Morita T., Fujiwara T., Yoshida A., Uotani K., Kiyono M., Yokoo S., Hasei J., Kunisada T., Ozaki T. (2020). Clinical relevance and functional significance of cell-free microRNA-1260b expression profiles in infiltrative myxofibrosarcoma. Sci. Rep..

[49] Gong L., Bao Q., Hu C., Wang J., Zhou Q., Wei L., Tong L., Zhang W., Shen Y. (2018). Exosomal miR-675 from metastatic osteosarcoma promotes cell migration and invasion by targeting CALN1. Biochem. Biophys. Res. Commun..

[50] Wu C., Li Z., Feng G., Wang L., Xie J., Jin Y., Liu S. (2021). Tumor suppressing role of serum-derived exosomal microRNA-15a in osteosarcoma cells through the GATA binding protein 2/murine double minute 2 axis and the p53 signaling pathway. Bioengineered.

[51] Ghamloush F., Ghayad S.E., Rammal G., Fahs A., Ayoub A.J., Merabi Z., Harajly M., Zalzali H., Saab R. (2019). The PAX3-FOXO1 oncogene alters exosome miRNA content and leads to paracrine effects mediated by exosomal miR-486. Sci. Rep..

[52] Casadei L., Calore F., Creighton C.J., Guescini M., Batte K., Iwenofu O.H., Zewdu A., Braggio D.A., Bill K.L., Fadda P. (2017). Exosome-Derived miR-25-3p and miR-92a-3p Stimulate Liposarcoma Progression. Cancer Res..

[53] Tian Q., Jia J., Ling S., Liu Y., Yang S., Shao Z. (2014). A causal role for circulating miR-34b in osteosarcoma. Eur. J. Surg. Oncol..

[54] Cong C., Wang W., Tian J., Gao T., Zheng W., Zhou C. (2018). Identification of serum miR-124 as a biomarker for diagnosis and prognosis in osteosarcoma. Cancer Biomark..

[55] Zhou L., Ma X., Yue J., Chen T., Wang X.Y., Wang Z.W., Pan J., Lin Y. (2018). The diagnostic effect of serum miR-139-5p as an indicator in osteosarcoma. Cancer Biomark..

[56] Yao Z.S., Li C., Liang D., Jiang X.B., Tang J.J., Ye L.Q., Yuan K., Ren H., Yang Z.D., Jin D.X. (2018). Diagnostic and prognostic implications of serum miR-101 in osteosarcoma. Cancer Biomark..

[57] Liu W., Zhao X., Zhang Y.J., Fang G.W., Xue Y. (2018). MicroRNA-375 as a potential serum biomarker for the diagnosis, prognosis, and chemosensitivity prediction of osteosarcoma. J. Int. Med. Res..

[58] Cai H., Zhao H., Tang J., Wu H. (2015). Serum miR-195 is a diagnostic and prognostic marker for osteosarcoma. J. Surg. Res..

[59] Allen-Rhoades W., Kurenbekova L., Satterfield L., Parikh N., Fuja D., Shuck R.L., Rainusso N., Trucco M., Barkauskas D.A., Jo E. (2015). Cross-species identification of a plasma microRNA signature for detection, therapeutic monitoring, and prognosis in osteosarcoma. Cancer Med..

[60] Pang P.C., Shi X.Y., Huang W.L., Sun K. (2016). miR-497 as a potential serum biomarker for the diagnosis and prognosis of osteosarcoma. Eur. Rev. Med. Pharmacol. Sci..

[61] Niu J., Sun Y., Guo Q., Niu D., Liu B. (2016). Serum miR-95-3p is a diagnostic and prognostic marker for osteosarcoma. SpringerPlus.

[62] Zhao X., Yang Y., Xu J., Luo Y., Xin Y., Wang Y. (2018). Downregulation of microRNA-95-3p suppresses cell growth of osteosarcoma via CDKN1A/p21 expression. Oncol. Rep..

[63] Yuan S., Wu Q., Wang Z., Che Y., Zheng S., Chen Y., Zhong X., Shi F. (2021). miR-223: An Immune Regulator in Infectious Disorders. Front. Immunol..

[64] Dong J., Liu Y., Liao W., Liu R., Shi P., Wang L. (2016). miRNA-223 is a potential diagnostic and prognostic marker for osteosarcoma. J. Bone Oncol..

[65] Wang S.N., Luo S., Liu C., Piao Z., Gou W., Wang Y., Guan W., Li Q., Zou H., Yang Z.Z. (2017). miR-491 Inhibits Osteosarcoma Lung Metastasis and Chemoresistance by Targeting αB-crystallin. Mol. Ther..

[66] Shi L., Xie C., Zhu J., Chen X. (2020). Downregulation of serum miR-194 predicts poor prognosis in osteosarcoma patients. Ann. Diagn. Pathol..

[67] Novello C., Pazzaglia L., Cingolani C., Conti A., Quattrini I., Manara M.C., Tognon M., Picci P., Benassi M.S. (2013). miRNA expression profile in human osteosarcoma: Role of miR-1 and miR-133b in proliferation and cell cycle control. Int. J. Oncol..

[68] Zhang C., Yao C., Li H., Wang G., He X. (2014). Serum levels of microRNA-133b and microRNA-206 expression predict prognosis in patients with osteosarcoma. Int. J. Clin. Exp. Pathol..

[69] Luo Z., Liu M., Zhang H., Xia Y. (2016). Association of circulating miR-125b and survival in patients with osteosarcoma-A single center experience. J. Bone Oncol..

[70] Nie C.L., Ren W.H., Ma Y., Xi J.S., Han B. (2015). Circulating miR-125b as a biomarker of Ewing’s sarcoma in Chinese children. Genet. Mol. Res..

[71] Tombolan L., Millino C., Pacchioni B., Cattelan M., Zin A., Bonvini P., Bisogno G. (2020). Circulating miR-26a as Potential Prognostic Biomarkers in Pediatric Rhabdomyosarcoma. Front. Genet..

[72] Tong X., Wang X., Wang C., Li L. (2018). Elevated levels of serum MiR-152 and miR-24 in uterine sarcoma: Potential for inducing autophagy via SIRT1 and deacetylated LC3. Br. J. Biomed. Sci..

[73] Batista P.J., Chang H.Y. (2013). Long noncoding RNAs: Cellular address codes in development and disease. Cell.

[74] Song Z., Lin J., Li Z., Huang C. (2021). The nuclear functions of long noncoding RNAs come into focus. Non-Coding RNA Res..

[75] Uszczynska-Ratajczak B., Lagarde J., Frankish A., Guigó R., Johnson R. (2018). Towards a complete map of the human long non-coding RNA transcriptome. Nat. Rev. Genet..

[76] Qian Y., Shi L., Luo Z. (2020). Long Non-coding RNAs in Cancer: Implications for Diagnosis, Prognosis, and Therapy. Front. Med..

[77] Moonmuang S., Chaiyawat P., Jantrapirom S., Pruksakorn D., Lo Piccolo L. (2021). Circulating Long Non-Coding RNAs as Novel Potential Biomarkers for Osteogenic Sarcoma. Cancers.

[78] Sheng K., Li Y. (2019). LncRNA TUG1 promotes the development of osteosarcoma through RUNX2. Exp. Ther. Med..

[79] Ma B., Li M., Zhang L., Huang M., Lei J.B., Fu G.H., Liu C.X., Lai Q.W., Chen Q.Q., Wang Y.L. (2016). Upregulation of long non-coding RNA TUG1 correlates with poor prognosis and disease status in osteosarcoma. Tumour. Biol..

[80] Cai L., Lv J., Zhang Y., Li J., Wang Y., Yang H. (2017). The lncRNA HNF1A-AS1 is a negative prognostic factor and promotes tumorigenesis in osteosarcoma. J. Cell Mol. Med..

[81] Wang Y., Zhao Z., Zhang S., Li Z., Li D., Yang S., Zhang H., Zeng X., Liu J. (2018). LncRNA FAL1 is a negative prognostic biomarker and exhibits pro-oncogenic function in osteosarcoma. J. Cell. Biochem..

[82] Chen S., Liu Z., Lu S., Hu B. (2019). EPEL promotes the migration and invasion of osteosarcoma cells by upregulating ROCK1. Oncol. Lett..

[83] Huo Y., Li Q., Wang X., Jiao X., Zheng J., Li Z., Pan X. (2017). MALAT1 predicts poor survival in osteosarcoma patients and promotes cell metastasis through associating with EZH2. Oncotarget.

[84] Malakoti F., Targhazeh N., Karimzadeh H., Mohammadi E., Asadi M., Asemi Z., Alemi F. (2022). Multiple function of lncRNA MALAT1 in cancer occurrence and progression. Chem. Biol. Drug Des..

[85] Song Q.H., Guo M.J., Zheng J.S., Zheng X.H., Ye Z.H., Wei P. (2020). Study on Targeting Relationship Between miR-320b and FGD5-AS1 and Its Effect on Biological Function of Osteosarcoma Cells. Cancer Manag. Res..

[86] Arroyo J.D., Chevillet J.R., Kroh E.M., Ruf I.K., Pritchard C.C., Gibson D.F., Mitchell P.S., Bennett C.F., Pogosova-Agadjanyan E.L., Stirewalt D.L. (2011). Argonaute2 complexes carry a population of circulating microRNAs independent of vesicles in human plasma. Proc. Natl. Acad. Sci. USA.

[87] Vickers K.C., Palmisano B.T., Shoucri B.M., Shamburek R.D., Remaley A.T. (2011). MicroRNAs are transported in plasma and delivered to recipient cells by high-density lipoproteins. Nat. Cell Biol..

[88] Bayraktar R., Van Roosbroeck K., Calin G.A. (2017). Cell-to-cell communication: MicroRNAs as hormones. Mol. Oncol..

[89] Han F., Wang C., Wang Y., Zhang L. (2017). Long noncoding RNA ATB promotes osteosarcoma cell proliferation, migration and invasion by suppressing miR-200s. Am. J. Cancer Res..

[90] Li W., Xie P., Ruan W.H. (2016). Overexpression of lncRNA UCA1 promotes osteosarcoma progression and correlates with poor prognosis. J. Bone Oncol..

[91] Wen J.J., Ma Y.D., Yang G.S., Wang G.M. (2017). Analysis of circulating long non-coding RNA UCA1 as potential biomarkers for diagnosis and prognosis of osteosarcoma. Eur. Rev. Med. Pharmacol. Sci..

[92] Zhao B., Liu K., Cai L. (2019). LINK-A lncRNA functions in the metastasis of osteosarcoma by upregulating HIF1α. Oncol. Lett..

[93] Jiang Y., Luo Y. (2020). LINC01354 Promotes Osteosarcoma Cell Invasion by Up-regulating Integrin β1. Arch. Med. Res..

[94] Zhang G.F., Zhou B.S., An X.C., An F.M., Li S.H. (2021). LINC01278 is Highly Expressed in Osteosarcoma and Participates in the Development of Tumors by Mediating the miR-134-5p/KRAS Axis. Onco Targets Ther..

[95] Cheng C., Zhang Z., Cheng F., Shao Z. (2020). Exosomal lncRNA RAMP2-AS1 Derived from Chondrosarcoma Cells Promotes Angiogenesis through miR-2355-5p/VEGFR2 Axis. Onco Targets Ther..

[96] Chen S., Xu X., Lu S., Hu B. (2019). Long non-coding RNA HAND2-AS1 targets glucose metabolism and inhibits cancer cell proliferation in osteosarcoma. Oncol. Lett..

[97] Yang Q., Yu H., Yin Q., Hu X., Zhang C. (2019). lncRNA-NEF is downregulated in osteosarcoma and inhibits cancer cell migration and invasion by downregulating miRNA-21. Oncol. Lett..

[98] Liu H., Zhou G., Fu X., Cui H., Pu G., Xiao Y., Sun W., Dong X., Zhang L., Cao S. (2017). Long noncoding RNA TUG1 is a diagnostic factor in lung adenocarcinoma and suppresses apoptosis via epigenetic silencing of BAX. Oncotarget.

[99] Alkhathami A.G., Hadi A., Alfaifi M., Alshahrani M.Y., Verma A.K., Beg M.M.A. (2022). Serum-Based lncRNA ANRIL, TUG1, UCA1, and HIT Expressions in Breast Cancer Patients. Dis. Markers.

[100] Yin Q., Shen X., Cui X., Ju S. (2019). Elevated serum lncRNA TUG1 levels are a potential diagnostic biomarker of multiple myeloma. Exp. Hematol..

[101] Tong Y.S., Wang X.W., Zhou X.L., Liu Z.H., Yang T.X., Shi W.H., Xie H.W., Lv J., Wu Q.Q., Cao X.F. (2015). Identification of the long non-coding RNA POU3F3 in plasma as a novel biomarker for diagnosis of esophageal squamous cell carcinoma. Mol. Cancer.

[102] Zhu C.H., Xiao D.H., Dai L.G., Xu H.G., Jiang Y.H., Zheng Z.J. (2018). Highly expressed lncRNA FAL1 promotes the progression of gastric cancer by inhibiting PTEN. Eur. Rev. Med. Pharmacol. Sci..

[103] Li B., Mao R., Liu C., Zhang W., Tang Y., Guo Z. (2018). LncRNA FAL1 promotes cell proliferation and migration by acting as a CeRNA of miR-1236 in hepatocellular carcinoma cells. Life Sci..

[104] Miao Y., Fan R., Chen L., Qian H. (2016). Clinical Significance of Long Non-coding RNA MALAT1 Expression in Tissue and Serum of Breast Cancer. Ann. Clin. Lab. Sci..

[105] Liu N., Feng S., Li H., Chen X., Bai S., Liu Y. (2020). Long non-coding RNA MALAT1 facilitates the tumorigenesis, invasion and glycolysis of multiple myeloma via miR-1271-5p/SOX13 axis. J. Cancer Res. Clin. Oncol..

[106] Lu Z., Luo T., Pang T., Du Z., Yin X., Cui H., Fang G., Xue X. (2019). MALAT1 promotes gastric adenocarcinoma through the MALAT1/miR-181a-5p/AKT3 axis. Open Biol..

[107] Zhang R., Xia Y., Wang Z., Zheng J., Chen Y., Li X., Wang Y., Ming H. (2017). Serum long non coding RNA MALAT-1 protected by exosomes is up-regulated and promotes cell proliferation and migration in non-small cell lung cancer. Biochem. Biophys. Res. Commun..

[108] Chen W., Xu X.K., Li J.L., Kong K.K., Li H., Chen C., He J., Wang F., Li P., Ge X.S. (2017). MALAT1 is a prognostic factor in glioblastoma multiforme and induces chemoresistance to temozolomide through suppressing miR-203 and promoting thymidylate synthase expression. Oncotarget.

[109] Qiu J.J., Lin X.J., Tang X.Y., Zheng T.T., Lin Y.Y., Hua K.Q. (2018). Exosomal Metastasis-Associated Lung Adenocarcinoma Transcript 1 Promotes Angiogenesis and Predicts Poor Prognosis in Epithelial Ovarian Cancer. Int. J. Biol. Sci..

[110] Liu B., Chen J., Shang F., Lian M., Shen X., Fang J. (2022). Tumor-Derived Exosome FGD5-AS1 Promotes Angiogenesis, Vascular Permeability, and Metastasis in Thyroid Cancer by Targeting the miR-6838-5p/VAV2 Axis. J. Oncol..

[111] Abedini P., Fattahi A., Agah S., Talebi A., Beygi A.H., Amini S.M., Mirzaei A., Akbari A. (2019). Expression analysis of circulating plasma long noncoding RNAs in colorectal cancer: The relevance of lncRNAs ATB and CCAT1 as potential clinical hallmarks. J. Cell Physiol..

[112] Lee Y.R., Kim G., Tak W.Y., Jang S.Y., Kweon Y.O., Park J.G., Lee H.W., Han Y.S., Chun J.M., Park S.Y. (2019). Circulating exosomal noncoding RNAs as prognostic biomarkers in human hepatocellular carcinoma. Int. J. Cancer.

[113] El-Ashmawy N.E., Hussien F.Z., El-Feky O.A., Hamouda S.M., Al-Ashmawy G.M. (2020). Serum LncRNA-ATB and FAM83H-AS1 as diagnostic/prognostic non-invasive biomarkers for breast cancer. Life Sci..

[114] Chang L., Xu W., Zhang Y., Gong F. (2019). Long non-coding RNA-NEF targets glucose transportation to inhibit the proliferation of non-small-cell lung cancer cells. Oncol. Lett..

[115] Liang Z., Zhu B., Meng D., Shen X., Li X., Wang Z., Li L. (2019). Down-regulation of lncRNA-NEF indicates poor prognosis in intrahepatic cholangiocarcinoma. Biosci. Rep..

[116] Wang X., Jiang X., Zhou L., Wang Z., Huang H., Wang M. (2019). LncRNA-NEF is involved the regulation of gastric carcinoma cell proliferation by targeting RUNX1. Mol. Med. Rep..

[117] Huang Q., Chen H., Zuo B., Cheng C., Yu W., Yang Y. (2019). lncRNA NEF inhibits glioma by downregulating TGF-β1. Exp. Ther. Med..

[118] Pan J., Xie X., Li H., Li Z., Ren C., Ming L. (2019). Detection of serum long non-coding RNA UCA1 and circular RNAs for the diagnosis of bladder cancer and prediction of recurrence. Int. J. Clin. Exp. Pathol..

[119] Zheng Z.K., Pang C., Yang Y., Duan Q., Zhang J., Liu W.C. (2018). Serum long noncoding RNA urothelial carcinoma-associated 1: A novel biomarker for diagnosis and prognosis of hepatocellular carcinoma. J. Int. Med. Res..

[120] Yu Y., Gao F., He Q., Li G., Ding G. (2020). lncRNA UCA1 Functions as a ceRNA to Promote Prostate Cancer Progression via Sponging miR143. Mol. Ther. Nucleic Acids.

[121] Wang M., Zhang Z., Pan D., Xin Z., Bu F., Zhang Y., Tian Q., Feng X. (2021). Circulating lncRNA UCA1 and lncRNA PGM5-AS1 act as potential diagnostic biomarkers for early-stage colorectal cancer. Biosci. Rep..

[122] Luan Y., Li X., Zhao R., Li Y., Liu L., Hao Y., Oleg Vladimir B., Jia L. (2020). Circulating lncRNA UCA1 Promotes Malignancy of Colorectal Cancer via the miR-143/MYO6 Axis. Mol. Ther. Nucleic Acids.

[123] Zhang H., Yao B., Tang S., Chen Y. (2019). LINK-A Long Non-Coding RNA (lncRNA) Participates in Metastasis of Ovarian Carcinoma and Upregulates Hypoxia-Inducible Factor 1 (HIF1α). Med. Sci. Monit..

[124] Ashwal-Fluss R., Meyer M., Pamudurti N.R., Ivanov A., Bartok O., Hanan M., Evantal N., Memczak S., Rajewsky N., Kadener S. (2014). circRNA biogenesis competes with pre-mRNA splicing. Mol. Cell.

[125] Starke S., Jost I., Rossbach O., Schneider T., Schreiner S., Hung L.H., Bindereif A. (2015). Exon circularization requires canonical splice signals. Cell Rep..

[126] Liang D., Tatomer D.C., Luo Z., Wu H., Yang L., Chen L.L., Cherry S., Wilusz J.E. (2017). The Output of Protein-Coding Genes Shifts to Circular RNAs When the Pre-mRNA Processing Machinery Is Limiting. Mol. Cell.

[127] Jeck W.R., Sorrentino J.A., Wang K., Slevin M.K., Burd C.E., Liu J., Marzluff W.F., Sharpless N.E. (2013). Circular RNAs are abundant, conserved, and associated with ALU repeats. RNA.

[128] Conn S.J., Pillman K.A., Toubia J., Conn V.M., Salmanidis M., Phillips C.A., Roslan S., Schreiber A.W., Gregory P.A., Goodall G.J. (2015). The RNA binding protein quaking regulates formation of circRNAs. Cell.

[129] Salzman J., Gawad C., Wang P.L., Lacayo N., Brown P.O. (2012). Circular RNAs are the predominant transcript isoform from hundreds of human genes in diverse cell types. PLoS ONE.

[130] Salzman J., Chen R.E., Olsen M.N., Wang P.L., Brown P.O. (2013). Cell-type specific features of circular RNA expression. PLoS Genet..

[131] Enuka Y., Lauriola M., Feldman M.E., Sas-Chen A., Ulitsky I., Yarden Y. (2016). Circular RNAs are long-lived and display only minimal early alterations in response to a growth factor. Nucleic Acids Res..

[132] Zheng Q., Bao C., Guo W., Li S., Chen J., Chen B., Luo Y., Lyu D., Li Y., Shi G. (2016). Circular RNA profiling reveals an abundant circHIPK3 that regulates cell growth by sponging multiple miRNAs. Nat. Commun..

[133] Li B., Zhu L., Lu C., Wang C., Wang H., Jin H., Ma X., Cheng Z., Yu C., Wang S. (2021). circNDUFB2 inhibits non-small cell lung cancer progression via destabilizing IGF2BPs and activating anti-tumor immunity. Nat. Commun..

[134] Du W.W., Yang W., Liu E., Yang Z., Dhaliwal P., Yang B.B. (2016). Foxo3 circular RNA retards cell cycle progression via forming ternary complexes with p21 and CDK2. Nucleic Acids Res..

[135] Wu N., Yuan Z., Du K.Y., Fang L., Lyu J., Zhang C., He A., Eshaghi E., Zeng K., Ma J. (2019). Translation of yes-associated protein (YAP) was antagonized by its circular RNA via suppressing the assembly of the translation initiation machinery. Cell Death Differ..

[136] Abe N., Matsumoto K., Nishihara M., Nakano Y., Shibata A., Maruyama H., Shuto S., Matsuda A., Yoshida M., Ito Y. (2015). Rolling Circle Translation of Circular RNA in Living Human Cells. Sci. Rep..

[137] Gao X., Xia X., Li F., Zhang M., Zhou H., Wu X., Zhong J., Zhao Z., Zhao K., Liu D. (2021). Circular RNA-encoded oncogenic E-cadherin variant promotes glioblastoma tumorigenicity through activation of EGFR-STAT3 signalling. Nat. Cell. Biol..

[138] Meyer K.D., Patil D.P., Zhou J., Zinoviev A., Skabkin M.A., Elemento O., Pestova T.V., Qian S.B., Jaffrey S.R. (2015). 5′ UTR m(6)A Promotes Cap-Independent Translation. Cell.

[139] Kristensen L.S., Jakobsen T., Hager H., Kjems J. (2022). The emerging roles of circRNAs in cancer and oncology. Nat. Rev. Clin. Oncol..

[140] Vo J.N., Cieslik M., Zhang Y., Shukla S., Xiao L., Wu Y.M., Dhanasekaran S.M., Engelke C.G., Cao X., Robinson D.R. (2019). The Landscape of Circular RNA in Cancer. Cell.

[141] Wang S., Zhang K., Tan S., Xin J., Yuan Q., Xu H., Xu X., Liang Q., Christiani D.C., Wang M. (2021). Circular RNAs in body fluids as cancer biomarkers: The new frontier of liquid biopsies. Mol. Cancer.

[142] Kun-Peng Z., Chun-Lin Z., Jian-Ping H., Lei Z. (2018). A novel circulating hsa_circ_0081001 act as a potential biomarker for diagnosis and prognosis of osteosarcoma. Int. J. Biol. Sci..

[143] Zhu K., Niu L., Wang J., Wang Y., Zhou J., Wang F., Cheng Y., Zhang Q., Li H. (2019). Circular RNA hsa_circ_0000885 Levels are Increased in Tissue and Serum Samples from Patients with Osteosarcoma. Med. Sci. Monit..

[144] Wang Y., Liu J., Ma J., Sun T., Zhou Q., Wang W., Wang G., Wu P., Wang H., Jiang L. (2019). Exosomal circRNAs: Biogenesis, effect and application in human diseases. Mol. Cancer.

[145] Li Y., Zheng Q., Bao C., Li S., Guo W., Zhao J., Chen D., Gu J., He X., Huang S. (2015). Circular RNA is enriched and stable in exosomes: A promising biomarker for cancer diagnosis. Cell Res..

[146] Pan Y., Lin Y., Mi C. (2021). Cisplatin-resistant osteosarcoma cell-derived exosomes confer cisplatin resistance to recipient cells in an exosomal circ_103801-dependent manner. Cell Biol. Int..

[147] Li S., Pei Y., Wang W., Liu F., Zheng K., Zhang X. (2020). Extracellular nanovesicles-transmitted circular RNA has_circ_0000190 suppresses osteosarcoma progression. J. Cell Mol. Med..

[148] He C., Zheng S., Luo Y., Wang B. (2018). Exosome Theranostics: Biology and Translational Medicine. Theranostics.

[149] Endzeliņš E., Berger A., Melne V., Bajo-Santos C., Soboļevska K., Ābols A., Rodriguez M., Šantare D., Rudņickiha A., Lietuvietis V. (2017). Detection of circulating miRNAs: Comparative analysis of extracellular vesicle-incorporated miRNAs and cell-free miRNAs in whole plasma of prostate cancer patients. BMC Cancer.

[150] Wang X., Zhang H., Yang H., Bai M., Ning T., Deng T., Liu R., Fan Q., Zhu K., Li J. (2020). Exosome-delivered circRNA promotes glycolysis to induce chemoresistance through the miR-122-PKM2 axis in colorectal cancer. Mol. Oncol..

[151] Xin R., Gao Y., Wang R., Kadash-Edmondson K.E., Liu B., Wang Y., Lin L., Xing Y. (2021). isoCirc catalogs full-length circular RNA isoforms in human transcriptomes. Nat. Commun..

[152] Zhang J., Hou L., Zuo Z., Ji P., Zhang X., Xue Y., Zhao F. (2021). Comprehensive profiling of circular RNAs with nanopore sequencing and CIRI-long. Nat. Biotechnol..

[153] Liu C., Kannisto E., Yu G., Yang Y., Reid M.E., Patnaik S.K., Wu Y. (2020). Non-invasive Detection of Exosomal MicroRNAs via Tethered Cationic Lipoplex Nanoparticles (tCLN) Biochip for Lung Cancer Early Detection. Front. Genet..

[154] Noren Hooten N., Byappanahalli A.M., Vannoy M., Omoniyi V., Evans M.K. (2022). Influences of age, race, and sex on extracellular vesicle characteristics. Theranostics.

[155] Dluzen D.F., Noren Hooten N., Evans M.K. (2017). Extracellular RNA in aging. Wiley Interdiscip. Rev. RNA.

